# Cascaded Cross-Modality Fusion Network for 3D Object Detection

**DOI:** 10.3390/s20247243

**Published:** 2020-12-17

**Authors:** Zhiyu Chen, Qiong Lin, Jing Sun, Yujian Feng, Shangdong Liu, Qiang Liu, Yimu Ji, He Xu

**Affiliations:** 1 School of Computer Science, Nanjing University of Posts and Telecommunications, No. 9 Wenyuan Road, Yadong New District, Nanjing 210023, China; 2020070133@njupt.edu.cn (Z.C.); 2019070116@njupt.edu.cn (J.S.); 2020070134@njupt.edu.cn (Y.F.); lsd@njupt.edu.cn (S.L.); liuqiang@njupt.edu.cn (Q.L.); xuhe@njupt.edu.cn (H.X.); 2 College of Automation, Nanjing University of Posts and Telecommunications, No. 9 Wenyuan Road, Yadong New District, Nanjing 210023, China; 1018051307@njupt.edu.cn

**Keywords:** 3D object detection, point cloud processing, multi-sensor fusion, LIDAR

## Abstract

We focus on exploring the LIDAR-RGB fusion-based 3D object detection in this paper. This task is still challenging in two aspects: (1) the difference of data formats and sensor positions contributes to the misalignment of reasoning between the semantic features of images and the geometric features of point clouds. (2) The optimization of traditional IoU is not equal to the regression loss of bounding boxes, resulting in biased back-propagation for non-overlapping cases. In this work, we propose a cascaded cross-modality fusion network (CCFNet), which includes a cascaded multi-scale fusion module (CMF) and a novel center 3D IoU loss to resolve these two issues. Our CMF module is developed to reinforce the discriminative representation of objects by reasoning the relation of corresponding LIDAR geometric capability and RGB semantic capability of the object from two modalities. Specifically, CMF is added in a cascaded way between the RGB and LIDAR streams, which selects salient points and transmits multi-scale point cloud features to each stage of RGB streams. Moreover, our center 3D IoU loss incorporates the distance between anchor centers to avoid the oversimple optimization for non-overlapping bounding boxes. Extensive experiments on the KITTI benchmark have demonstrated that our proposed approach performs better than the compared methods.

## 1. Introduction

Among various tasks of scene understanding, object detection is crucial for autonomous driving [1], robotics, and augmented reality. Deep learning-based 2D object detection which aims to predict the position and category of targets with given images has made unprecedented achievement in recent years [2]. RGB images provide fine-grained contextual information but still lack accurate depth information, which lets the prediction of 2D object detection suffer from space ambiguity [3]. Recently, extensive research has focused on 3D object detection to estimate the accurate 3D location of the target, benefitting from available point cloud sources.

LIDAR provides spatial and geometric descriptions for the 3D environment which targets exist in, but point cloud still lacks texture and color information like RGB images. Therefore, LIDAR-RGB fusion based 3D object detection takes advantage of two sensors to compensate the weaknesses of each other and capture more discriminative features of objects. However, two distinct modalities with various data formats and properties lead to challenges in this task. RGB images have ordered and grid structure which has been studied in numerous research, while the point cloud has unordered and spare structure. Moreover, the problem of how to correlate the semantic features of images with the geometric features of point cloud is indispensable in the fusion process. In common sense, the semantic and contextual information of images is always extracted in high-level features and the shape and texture information always exists in low-level features [4]. These changing encoding characteristics make each stage of LIDAR-RGB fusion have specific demand and cooperation manner. For instance, in the low-level feature of images, the apparent texture and accurate shape information could easier match the geometric outlook of objects. In the deeper layers, the semantic and contextual features of images need implicit category-wise geometric information. To solve these problems, existing works mainly depend on cross-modality feature alignment to fuse the RGB and LIDAR features.

According to the way of fusing multi-modality sensor data, we classify previous works into three categories: (1) early fusion-based methods (2) late fusion-based methods and (3) deep fusion-based methods. For detail, early fusion-based methods usually utilize a separate perception algorithm to process the multi-modality raw sensor data. However, they always require the precise alignment of data. If the raw sensor data are not well aligned in the early stage, it would lead to heavy performance degradation due to the feature dislocation. Depending on coordinate location of two sensors, PointPainting [5] and PI-RCNN [6] project the image semantic segmentation to point cloud space by projecting matrix. Although this early fusion process enables the network to handle aligned two-modality information as a whole without specific modality adjustment, the early stage fusion also conveys the noise in one modality to another modality. This noise would unavoidably be aligned and combined with discriminative features of objects, significantly damaging the prominence of features.

Late fusion-based methods only fuse the processed features at the decision level because the spatial and modal difference between the point cloud and the image is greatly reduced in this stage. MV3D [7], AVOD [3], CLOCs [8] extract point cloud and image features through independent modules and fuses them at the decision-making layer. However, the fusion in the decision-making layer has little effect on the raw data information fusion, and the confidence scores of the proposals generated by the two modules are not related. For the deep fusion-based methods, 3D-CVF [9] and MMF [10] adopt feature extractors respectively for LIDAR and image, and fuse images and LIDAR hierarchically and semantically. Finally, the semantic fusion of multi-scale information is realized. However, these methods are difficult to solve the problems of the difference between data formats and sensor positions. Moreover, 3D-CVF lacks continuous feature fusion in the feature extraction process will result in insufficient feature fusion. MMF only utilizes the sparse depth map projected from the point cloud, which leads to a weaker influence of the point cloud data on the generation of the anchor.

To solve these challenges, we observe that it is hard to align two-modality features throwaway. As aforementioned, features with different characteristics always need corresponding features from another modality. However, this demand is unknown for hand-crafted fusion design. Moreover, the processes of encoding RGB and LIDAR always have the dynamic appetite for extracting specific features, e.g., the contextual features of images are in the deeper layers, while low-level features are in early layers. The features in which layers are suitable for feature alignment are changing in optimization. Therefore, it is more reasonable to build a dynamic multi-modal fusion method. In this paper, we propose a cascaded cross-modality fusion network (CCFNet) for LIDAR-RGB fusion-based 3D object detection to address the above challenges. Our CCFNet is developed to establish a dynamic alignment manner by letting each stage choose specific salient features from previous stages. Our CCFNet mainly consists of a cascaded multi-scale fusion module (CMF) and a novel center 3D IoU loss.

In order to build a dynamic aligned network, we insert cascaded multi-scale fusion module (CMF) between each stage of LIDAR and RGB streams. Our CMF collects point cloud features from adjacent stages and aligns them with image features. By processing CMF in a cascaded way, the alignment in each stage could adjustably select specific point cloud features from previous stages to meet its demand. Besides, as pointed in [11], traditional IoU loss has a plateau, making it infeasible to optimize in the case of nonoverlapping bounding boxes, which is much more severe in 3D cases. These non-overlapping anchors are still useful to let aligned RoI have a roughly location sensitivity, i.e., guiding the RPN to generate anchors close to true bounding boxes. In this work, we advocate a novel center 3D IoU loss to make use of this benefit of non-overlapping bounding boxes. By introducing the measurement of distance between anchor center and ground truth center, our center 3D IoU loss is equipped with the ability to decrease the possibility of unreasonable anchors.

Our contribution can be summarized as follows:

(1) We propose a novel cascade approach to fuse and align LIDAR-RGB information. Our approach obtains multiple residual operations which could back-propagate gradient of guidance of alignment to the previous parts in encoder to select informative point cloud features.

(2) In order to make use of non-overlapping bounding boxes, we propose a novel center 3D IoU loss to allow the model to be sensitive to the location of generated anchors.

(3) Our approach has achieved better performance on the KITTI benchmark and performs favorably compared to methods.

## 2. Related Work

### 2.1. RGB-Based 3D Object Detection

Recently, the performance of RGB-based 3D object detection is significantly improved because of mature deeper learning method, such as Faster-RCNN [12], SSD [13] and YOLO [14]. Song et al. [15] propose the 3D Region Proposal Network based on Faster R-CNN, which takes the 3D volume scene in the RGB-D image as input. This network finally outputs the bounding boxes of the 3D objects; Gupta et al. [16] change the network input to 2.5D (extracting suitable expression based on RGB-D), which can improve the speed of the object detection algorithm; Tekin [17] et al. are inspired by [12,14,18] to propose a new CNN, which predicts the location of the projected points of the target 3D bounding box in the 2D image domain. Finally, this method uses the camera pose estimation algorithm to predict the 6D pose of an object. Moreover, [13] proposes the method of synthesizing data and decomposing the pose space of the model is used to increase the detection rate, but if the target is occluded, the accuracy of the 3D bounding box will be greatly reduced. Furthermore, ref. [19] proposes the DenseFusion Network architecture, which fuses the depth of each pixel with the image information to infer the local fine appearance and geometric spatial information of the target to deal with the situation of heavy occlusion. Finally, DenseFusion integrates an iterative fine-tuning module in the neural network to improve the real-time processing speed.

### 2.2. LIDAR-Based 3D Object Detection

The works of 3D object detection algorithm based on LIDAR point cloud could be divided into: (1) pseudo-image based methods, (2) PointNet-based methods and (3) voxel-based methods. First of all, the pseudo-image-based methods take advantage of the expertise of 2D image understanding by projecting point cloud data to some specific angles of view, such as a bird’s-eye view and front view. These methods include VeloFCN [20], MV3D-LIDAR [7], PIXOR [21], PointRCNN [22], etc. However, the point cloud is sparse, which easily leads to loss of information in the projecting procedure [23]. Besides, Charles [24] et.al. propose the PointNet that can directly process point cloud data. It uses the MaxPooling symmetric function to extract point cloud features to solve the problem of disorder nature of point cloud. In this method, a small neural network is trained to ensure the invariance of the laser point cloud in the process of realizing rotation or translation conversion. However, the PointNet network simply connects all points and only considers global features and single point features, without local information, which results in poor results for multi-classification problems with multiple instances. To solve the above problems, Charles et al. [25] then propose an improved network Pointnet++, which obtains the deep semantic features of the target by cascading the processing modules of the sampling layer, combination layer, and feature extraction layer. In PointNet++, two strategies of multi-scale combination and multi-resolution combination are used to ensure more accurate target feature extraction. PV-RCNN [26] proposes a novel 3D object detection framework that deeply integrates both a 3D voxel convolutional neural network and a PointNet-based set abstraction. SA-SSD [27] is a point-based method, which improves accuracy by deeply mining the geometric information of three-dimensional objects. Moreover, 3DSSD [28] designs a new set abstraction module and discards the feature pyramid module to reduce inference time and training memory. Furthermore, [29] proposes VoxelNet, which divides the 3D point cloud into a certain number of voxels. First, VoxelNet performs random sampling and normalization of the point cloud to extract features for each non-empty voxel and obtains the geometric space representation of the target. Finally, VoxelNet uses RPN for classification and position regression. Voxel-FPN [30], SECOND [31], PointPillar [32] and Part-A^2^ [33] are all the same type of the voxel-based methods.

### 2.3. LIDAR-RGB Fusion-Based 3D Object Detection

The LIDAR-RGB 3D fusion object detection algorithm mainly includes MV3D [7], AVOD [3], 3D-CVF [9], MMF [10], etc., which are more robust in practical applications. MV3D is the first pioneer in using the fusion of LIDAR point cloud data and RGB image information. As a perceptual fusion framework, MV3D uses the front view (FV) and bird’s-eye view (BEV) of point cloud to represent 3D point cloud information and merges them with RGB images to predict directional 3D bounding box. In order to solve the problem of slow recognition speed of MV3D [3,7] proposes an AVOD fusion algorithm. AVOD first simplifies the input of MV3D and then improves the 3D RPN (region proposal network) architecture. Finally, it adds 3D bounding box geometric constraint improvements. On the basis of improving the recognition accuracy, the recognition rate of AVOD is significantly improved. Moreover, 3D-CVF generates dense RGB voxel features and uses the adaptive gated fusion network to align the RGB image with the LIDAR point cloud. Finally, cross-modal fusion is achieved through an accurate multi-modal data position.

## 3. Our Approach

**Figure 1:**
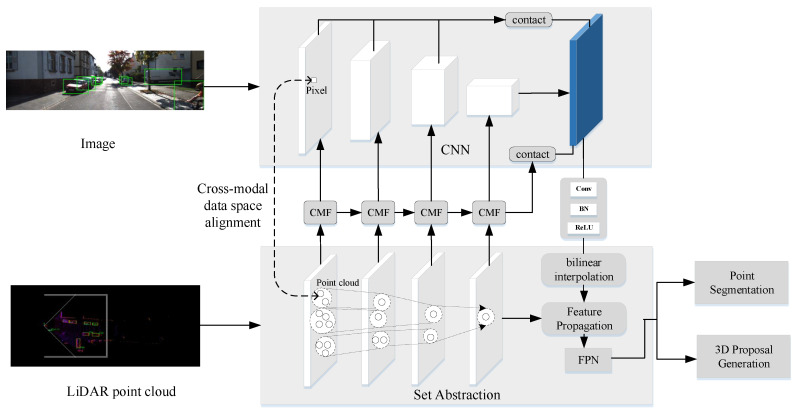
Overview of our CCFNet. ResNet50 and four Set Ablation modules of PointNet are used as feature extractors of RGB image and LIDAR respectively. Cascaded multi-scale fusion module (CMF) modules in four stages have little difference with each other.

In this paper, we propose a cascaded cross-modality fusion network (CCFNet) for LIDAR-RGB fusion-based 3D object detection. As shown in Figure 1, the features of LDIAR and RGB images are extracted by two separate streams. We use ResNet50 and four set ablation modules of PointNet as the feature extractor of RGB images and LIDAR respectively. Between each stage of two streams, we insert our cascaded multi-scale fusion module (CMF) to connect and fuse the image features and LIDAR features that share the same downsample ratio. Finally, the outputs of two streams are concatenated and sent to the detection head. We also describe the components of the total training losses, including our novel 3D IoU loss and the spatial setting of anchors and targets.

### 3.1. Cascaded Multi-Scale Fusion Module

We tackle the optimization of corresponding LIDAR-RGB feature fusion of each stage by building a cascaded structure where the image features of each stage could access the point cloud information from previous stages. In this way, the image features could dynamically select the suitable and multi-scale point cloud features from different stages and accordingly optimize the efficiency of LIDAR-RGB feature fusion.

Let i∈{1,2,3,4} denote the stage of LIDAR and RGB streams, $A_{i}\in R^{H\times W\times C^{a}}$ and $B_{i}\in R^{N\times C^{b}}$ are the RGB and LIDAR feature maps at the *i*-th stage, where $H,W,C^{a}$ denote the height, width and channel dimension of the image feature, and $N,C^{b}$ are the number of points and channel dimension of LIDAR feature respectively. Our CMF mainly has two main procedures, i.e., multi-scale fusion and LIDAR-RGB projecting. Since the CMF modules in different stages have little difference, we first introduce the general mechanism of our CMF module and then describe the actual implementation of CMF modules in different stages.

**Figure 2:**
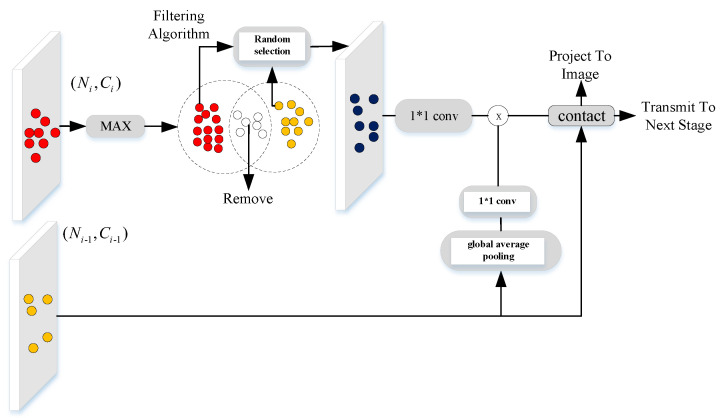
The general mechanism of our CMF module. This scheme consists of two main procedures: multi-scale fusion and LIDAR-RGB projecting.

As shown in Figure 2, the CMF module has two inputs, i.e., the feature $A_{i-1}$ from the previous stage and $A_{i}$ from the current stage. We first select the points having sailent features in $A_{i-1}$, which has $N_{i-1}$ points, and then fuse it with $A_{i}$. For detail, we first use max() function to highlight the category characteristic of feature and then select $N_{i}$ points possessing a larger value from the point set of $A_{i-1}$. According to the index of selected points, selected point features $A_{i-1}^{s}\in R^{N_{i}\times C_{i-1}^{b}}$ can be easily found. By processing one Conv1d, $A_{i-1}^{s}$ is activated by the global vector from $A_{i}$ by global average pooling, which finally generates $A_{i-1}^{gap}$. Then, $A_{i}^{cat}\in R^{2N_{i}\times C_{i}^{b}}$ is conducted after concating $A_{i}$ and $A_{i-1}^{gap}$ along the *N* dimension. We name, after this process, multi-scale fusion, which could be represented as follows:

(1) $$\vec{A_{i-1}}=max\left(A_{i-1}\right),$$

(2) $$A_{i-1}^{s}=\phi \left(\vec{A_{i-1}},A_{i-1},N_{i}\right),$$

(3) $$P=Tile(GlobalAveragePooling\left(A_{i}\right)),$$

(4) $$D=Sigmoid(||P-A_{i-1}{\left|\right|}_{2}-Mean(||P-{A||}_{2})),$$

(5) $$A_{i-1}^{gap}=Conv1d\left(A_{i-1}^{s}\right)\cdot D,$$

(6) $$A_{i}^{cat}=Concat\left(A_{i-1}^{gap},A_{i}\right),$$

where ϕ() denote the step of choosing $N_{i}$ points with salient features from $A_{i-1}$ and Tile() is the function of tiling the vector along *N* dimension to generate a tensor whose resolution is the same as $A_{i}$.

Besides, we project the point cloud features $A_{i}^{cat}$ to image space through the LIDAR-RGB projecting procedure. Specifically, we utilize the principle of spatial perspective to project LIDAR points to image. Each position $\left(x^{a},y^{a},z^{a}\right)$ of points *a* belonging to the point set of $A_{i}^{cat}$ should be multiplied by image size, since these coordinates of position along *X* and *Y* axes have been normalized to [−1,1]. Therefore, the corresponding position $\left(x^{m},y^{m}\right)$ of point *a* in image, where the superscript *m* denotes the image domain, could be calculated by:

(7) $$x_{m}=\frac{\left(x^{a}+1\right)\left(w_{i}-1\right)}{2},y_{m}=\frac{\left(y^{a}+1\right)\left(H_{i}-1\right)}{2}$$

Then, $A_{i}$ is transmitted to the next stage as one of the inputs of the next CMF module.

For the CFM module in the first stage, we directly project $A_{1}$ to image space of $B_{1}$. For the CMF module in the second stage, we follow the full processes described above. However, in the third and fourth stages, the standard processes of CMF module would lead to the biased saturation of some points, since inherited features from previous stages have numerous repeated points. They would account most of the point candidates which have salient features, if the selecting process is not well regulated. This phenomenon would become severe when the stage goes deeper. Therefore, we design a regulation algorithm to avoid the oversaturated problem, as shown in Algorithm 1. Moreover, the whole procedure of CMF in the third and fourth stages is shown in Figure 2.
**Algorithm 1** A regulation algorithm of CMF module to avoid over-saturation of repeated points in the points candidates.**Input:** point cloud features $A_{i-1}$ from previous stage and point set $S_{i-1}$ of $A_{i-1}$.**Output:** regulated point cloud features $A_{i-1}^{r}$.
1:Count the repeated points set $S_{i-1}^{m}$ and unrepeated points set $S_{i-1}^{n}$ in $S_{i-1}$ where $S_{i-1}^{m}\cup S_{i-1}^{n}=S_{i-1}$2:**if**$S_{i-1}^{m}$>$\lambda_{1}\cdot N_{i}$ or $S_{i-1}^{m}<\lambda_{2}\cdot N_{i}$ **then**3:    Random select $N_{i}$ points from $S_{i-1}$ and corresponding features $A_{i-1}^{r}$4:**else**5:    Random select $N_{i}$ points from $S_{i-1}^{m}$ to build a new repeated point set $S_{i-1}^{q}$6:    Random select $N_{i}$ points from $S_{i-1}^{q}\cup S_{i-1}^{n}$ and collect corresponding features $A_{i-1}^{r}$7:**end ifreturn**$A_{i-1}^{r}$

### 3.2. Center 3D IoU Loss

**Figure 3:**
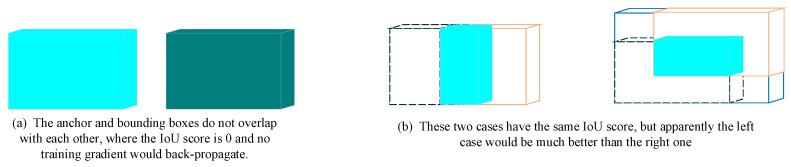
The illustration of non-overlapping case and inconsistcy between loss and the quality of obtained bouding boxes.

Moreover, the 3D object detector always generates abundant anchors to predict the true bounding boxes, whose number is much more than that of ground truth bounding boxes. However, due to the meaning of IoU and the setting of corresponding traditional IoU loss, only anchors which overlap with ground truth would contribute to the optimization, while those anchors which have no overlapping are simply punished and contribute nothing. As shown in Figure 3a, the anchor and bounding boxes do not overlap with each other, where the IoU score is 0 and no training gradient would back-propagate. However, the positive anchors only account a small part of total generated anchors, resulting in limited income compared with such a heavy computational cost. However, these non-overlapping anchors are still meaningful. We believe that even without overlapping, anchors which are near to ground truth bounding boxes are more useful than these anchors far from that. These non-overlapping anchors could provide a constant for RPN to build a position sensitivity which enables RPN to generate anchors close to true bounding boxes. Moreover, this distance information could adjust the inconsistency between loss and the quality of obtained bounding boxes. As shown in Figure 3b, these two cases have the same IoU score, but apparently the left case would be much better than the right one, i.e., the two axes are aligned in left, but one axis is aligned in right.

Inspired by this observation, we introduce our center 3D IoU loss to solve the aforementioned limitations. We define 3D anchor as (x,y,z,h,w,d,θ), where (x,y,z) is the 3D coordinate of anchor and bounding box center, h,w,d are the height, width and depth of 3D anchor, θ is the angle of anchor along *Z* axis. In order to calculate the IoU score of predicted anchor and ground truth bounding box which are denoted as superscript *p* and gt, we first obtain the whole 3D volume of predicted anchor $V^{an}$ and ground truth bounding box $V^{gt}$ and the overlapped volume of them $V^{overlap}$ and the volume of the smallest enclosing convex region $V^{scr}$. Therefore, 3D IoU could be formulated as:

(8) $$3D\;IoU=\frac{V^{overlap}}{V^{an}+V^{gt}-V^{overlap}}-\frac{|V^{scr}-\left(V^{an}+V^{gt}-V^{overlap}\right)|}{|V^{scr}|}$$

In order to revise the inconsistcy between loss and the quality of obtained bouding boxes as illustrated in Figure 3, we add the center distance 3Ddistance between anchor and ground truth bounding box:

(9) $$3D\;distance=\frac{\left|\right|x^{an}-x^{gt}{\left|\right|}_{2}+\left|\right|y^{an}-y^{gt}{\left|\right|}_{2}+\left|\right|z^{an}-z^{gt}{\left|\right|}_{2}}{\sqrt{\left|\right|h^{gt}{\left|\right|}_{2}+\left|\right|w^{gt}{\left|\right|}_{2}}}$$

Our center 3D IoU loss consists of these two parts and is formulated as follows:

(10) Center3DIoU=3DIoU+3Ddistance

This center 3D IoU loss optimizes the two IoU metrics: the overlapped area and the distance from the center of the anchor to that of ground truth bounding box. It helps the generator of anchors to be sensitive to the position of ground truth bounding box, since even without overlapping, the anchor near the ground truth also reduces the loss relatively. Then, the NMS process maintains a more accurate bounding box. The quantitative results and analysis in Section 4.4 indicate the effectiveness of our center 3DIOU loss in improving 3D detection performance.

### 3.3. 3D Region Proposal Network

We use a multibox SSD-like [31] RPN as detection head of our CCFNet. For the input of the 3D RPN, we use the feature maps generated by combining LIDAR and image features. Specifically, the architecture of RPN consists of three stages and each of them is composed of several convolutional layers and a downsampled convolutional layer sequentially. Then, the outputs of three stages are upsampled to a fixed size and are concatenated into one feature map. Finally, the concatenated feature map is sent to three 1×1 convolutions for classification and orientation estimation.

## 4. Experiments

### 4.1. Dataset and Evaluation Metrics

**KITTI**: The KITTI [34] records the details of scenery in both image and point cloud format. Moreover, 7481 training pairs and 7518 test pairs are included in three categories: car, cyclist, and pedestrian. According to the target size and distance, the difficulty of the data is divided into easy, moderate, and hard. Due to this, the ground truth of the test set is not available, so we follow the protocol of [7,29], splitting the training samples into a new train set and a new validation set. Our ablation study is conducted on a validation set and the final performance comparison with other research is conducted using test set obtained by server submission. The KITTI dataset uses the PR (precision recall) curve and AP value to judge the accuracy of the detection model. By setting different thresholds, different recall and precision are obtained to draw the PR curve. The 3D IoU evaluation thresholds are related to the target category. The official thresholds of car, cyclist, and pedestrian are 0.7, 0.5, and 0.5 by default.

### 4.2. Implementation Detail

Considering that the points of the distant objects in point cloud are too sparse to produce effective features, we select the point cloud in the range of [−40, 40] × [0, 70] × [ −1, 3] according to the X, Y, Z coordinate system. We extracted 16,382 point clouds from the raw point cloud data as the input of the point cloud feature extraction network and used a set abstraction layer to perform the input LIDAR point with the size of 4096,1024, 256, 64. The subsequent propagation layer was originally used for a point cloud semantic segmentation and 3D scheme generation, therefore we kept it in our network to maintain semantic information. We set the RGB image input resolution to 1280×384×3, and set up the up-sampling module and the contact module to fuse the image features of each step. The adam optimizer is adopted to optimize the network, which sets the initial learning efficiency to 0.002, the weight to 0.001, the training epoch to 50, and the batch size to 12. Our approach is implemented on single RTX 2080Ti, which has 11G memory.

### 4.3. Anchors and Targets

In this paper, we use the default setting of anchor for three categories as SECOND [31]. We assign a one-hot vector for anchor classification, 7 vectors for box regression, and a one-hot vector for direction classification. For the box regression, the following localization regression residuals are leveraged for training:

(11) $$\begin{array}{cc} \; & \Delta x=\frac{x^{gt}-x^{an}}{d^{an}},\Delta y=\frac{y^{gt}-y^{an}}{d^{an}},\Delta z=\frac{z^{gt}-z^{an}}{h^{an}},\\ \; & \Delta w=\log\frac{w^{gt}}{w^{an}},\Delta l=\log\frac{l^{gt}}{l^{an}},\Delta h=\log\frac{h^{gt}}{h^{an}},\\ \; & \Delta \theta =sin\left(\theta^{gt}-\theta^{an}\right),d^{an}=\sqrt{{\left(l^{an}\right)}^{2}+{\left(w^{an}\right)}^{2}}, \end{array}$$

where *x*, *y* and *z* are the 3D center coordinates; *w*, *l*, and *h* are the width, length and height of the 3D bounding box, respectively; θ is the yaw rotation around the up-axis (*z* axis); the superscript gt and an denote the ground truth and anchor respectively; and $d^{an}=\sqrt{{\left(l^{an}\right)}^{2}+{\left(w^{an}\right)}^{2}}$ is the diagonal line at the bottom of the anchor box. Our total loss is composed by the above losses and our center 3D IoU loss.

### 4.4. Ablation Study

In this section, we validate the effectiveness of the proposed CMF module and center 3D IoU loss. First, we insert our CMF module in different stages with 8 settings, as shown in Table 1. Compared with groups (1) to (5), it is obvious that cross-modality fusion performs better at a deeper stage. Especially in hard-level detection, the 3.11% improvement from no fusion to fusion in the fourth stage is a large margin. We believe that the reasoning of semantic information in images and the high-level geometric information in LIDAR, which could be aligned in easy level instances, further enhance the discriminative representation of instances in far distance. Moreover, we compare the groups (6)–(8) and notice that adding more connections between stages of image and LIDAR streams provides further improvement, indicating the cascaded and dense shortcuts between stages convey adjustable attention to choose the most suitable corresponding features in the fusion process. We also make extensive experiments on the setting of hyper-parameters of Algorithm 1. As shown in Table 2, we set the $\lambda_{1}$ in the range of [0.5,0.75] and $\lambda_{2}$ in the range of [0.75,0.85]. When the λ>0.6, keeping more repeated points in the point candidates would lead to a sharp decrease in performance. Similarly, the over-reducing of repeated points in point candidate would damage the effectiveness of selecting salient point features. Moreover, we evaluate our center 3D IoU loss and traditional IoU loss in Table 3. It is obvious that our center 3D IoU loss brings about significant improvement.

### 4.5. Evaluation on the KITTI Validation Set

**Figure 4:**
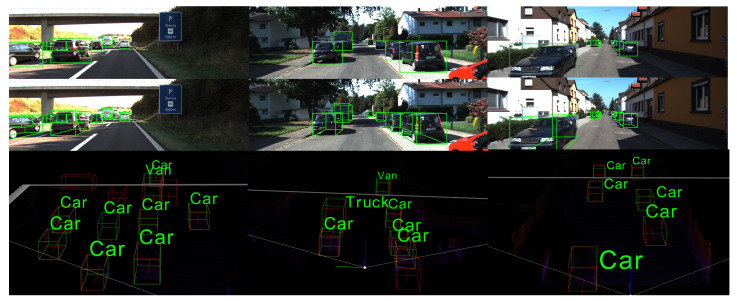
Qualitative performance of or approach on the KITTI validation set. The red 3D bounding boxes denote the ground truth bounding boxes and the green one is our predition.

We show the results evaluated on the KITTI validation set in Table 4 and Table 5 for the convenience of comparison with future work. Our network performs better than the method of two tables at all difficulty levels. We show some detection results in Figure 4. In order to facilitate visualization, we use 8 corner points of the 3D bounding box to visualize the 3D prediction. The red 3D bounding boxes denote the ground truth bounding boxes and the green one is our prediction. Overall, this evaluation shows that our proposed network can provide high-precision results.

### 4.6. Evaluation on the KITTI Test Set

In Table 6 and Table 7, we compare our approach with the previously proposed method on BEV and 3D dectection task. From the comparison results, we can conclude that our approach has a better effect on BEV and 3D detection tasks. Our approach is compared with other public algorithms, and the types of input data are divided into two categories: LIDAR and LIDAR + Image. We observe that the proposed CCFNet can achieve better performance than other compared LIDAR-RGB fusion-based and LIDAR-based methods. CCFNet outperforms VoxelNet by 13.16%, PointRCNN by 2.86%, SECOND by 4.96%, MV3D by 16.27%, AVOD by 12.84%, AVOD-FPN by 6.74%, FPointNet by 8.23%, MMF by 1.19%, CLOCs by 0.17% in terms of 3D middle-level mAP. Moreover, Table 7 shows the performance of our approach for 3D BEV object localization. CCFNet can achieve much better performance than other LIDAR-RGB fusion-based and LIDAR-based detection, outperforming VoxelNet by 9.36%, PointRCNN by 13.2%, SECOND by 14.96%, MV3D by 11.72%, AVOD by 3.18%, AVOD-FPN by 4.83%, F-PointNet by 4.62%, MMF by 0.41%, CLOCs_SecCas by 0.39% in terms of BEV middle-level mAP. The result in the KITTI leaderboard is available at: http://www.cvlibs.net/datasets/kitti/eval_object.php?obj_benchmark=3d.

## 5. Conclusions

In this paper, we propose a cascaded cross-modality fusion approach (CCFNet) to improve the LIDAR-RGB feature fusion process. We tackle the challenges of multi-sensor fusion as a dynamic matching problem, which meets the need of adaptively choosing suitable point cloud features from previous stages. Technically, on the one hand, our CMF module selects the points which have salient features to project to image space and are transmitted to the next stage. We insert our CMF module into each stage of LIDAR and RGB streams to build dynamic attention for meeting the demand for selecting corresponding point cloud features from different stages. On the other hand, a novel center IoU loss is proposed to make use of non-overlapping bounding boxes which account for most of the generated anchor candidates. It enables our network to generate anchors close to the ground truth bounding boxes, instead of generating unreasonable anchors. Extensive experiments have demonstrated the effectiveness of the CMF module and center IoU loss. We also achieve better performance than compared methods on the KITTI benchmark. Moreover, our approach still has some limitations. The proposed CMF modules unavoidably increase the computational costs, which are vital for real-world applications. Inspired by PVCNN [36], we will improve the point cloud feature extraction module in the next step.

## Figures and Tables

**Table 1:** Ablation study of CMF nodule in different stages. The results are reported on the KITTI validation set.

Group	Stage				3D Detection		
	1	2	3	4	Easy	Mod	Hard
(1)					87.82	77.41	75.94
(2)	*√*				88.47	78.68	76.10
(3)		*√*			89.72	78.60	76.51
(4)			*√*		89.25	78.92	76.91
(5)				*√*	89.35	79.10	77.03
(6)	*√*	*√*			90.02	79.90	78.63
(7)	*√*	*√*	*√*		90.80	80.19	78.73
(8)	*√*	*√*	*√*	*√*	90.93	81.79	79.60

**Table 2:** Ablation study of setting of $\lambda_{1}$ and $\lambda_{2}$ of Algorithm 1. The results are reported on the KITTI validation set.

$\lambda_{1}$	$\lambda_{2}$	3D Detection		
		Easy	Mod	Hard
0.5	0.75	90.23	80.21	79.22
0.5	0.85	90.01	80.13	79.09
0.6	0.75	90.41	79.58	78.82
0.6	0.85	89.23	79.25	78.53
0.75	0.85	89.14	78.91	78.22

**Table 3:** Ablation study of center 3D IoU loss nodule in different stages. The results are reported on the KITTI validation set.

Method	3D Detection		
	Easy	Mod	Hard
traditional IoU	88.41	80.21	78.21
CCF IoU	91.12	82.42	79.63

**Table 4:** Performance comparison with the compared methods on 3D car detection (AP, %) on the KITTI validation set.

Method	Easy	Mod	Hard
MV3D [7]	71.29	62.28	56.56
AVOD-FPN [3]	84.41	74.44	68.65
FPointNet [35]	83.76	70.92	63.65
SECOND [31]	87.43	76.48	69.10
CCFNet	91.93	81.79	79.60

**Table 5:** Performance comparison with the compared methods on BEV car detection (AP, %) on the KITTI validation set.

Method	Easy	Mod	Hard
MV3D [7]	86.55	78.10	76.67
AVOD-FPN [3]	84.41	74.44	68.65
FPointNet [35]	88.16	84.02	76.44
SECOND [31]	89.96	87.07	79.66
CCFNet	94.01	88.92	82.93

**Table 6:** Performance comparison with the compared methods on 3D object detection (AP, %) on the KITTI test set.

Method	Car			Pedestrian			Cyclist			Input Data
	Easy	Mod	Hard	Easy	Mod	Hard	Easy	Mod	Hard	
VoxelNet [29]	81.97	65.46	62.85	57.86	53.42	48.87	67.17	47.65	45.11	LIDAR
PointRCNN [22]	85.94	75.76	68.32	49.43	41.78	38.63	73.93	59.60	53.59	LIDAR
SECOND [31]	83.13	73.66	66.20	51.07	42.56	37.29	70.51	53.85	46.90	LIDAR
MV3D [7]	71.09	62.35	55.12	N/A	N/A	N/A	N/A	N/A	N/A	LIDAR + Image
AVOD [3]	73.59	65.78	58.38	38.28	31.51	26.98	60.11	44.90	38.80	LIDAR + Image
AVOD-FPN [3]	81.94	71.88	66.38	46.35	39.00	36.58	59.97	46.12	42.36	LIDAR + Image
FPointNet [35]	81.20	70.39	62.19	51.21	44.89	40.23	71.96	56.77	50.39	LIDAR + Image
MMF [10]	88.40	77.43	70.22	N/A	N/A	N/A	N/A	N/A	N/A	LIDAR + Image
CLOCs_SecCas [8]	86.38	78.45	72.45	N/A	N/A	N/A	N/A	N/A	N/A	LIDAR + Image
Our	89.07	78.62	73.33	53.92	46.58	42.14	73.65	57.26	52.42	LIDAR + Image

**Table 7:** Performance comparison with the compared methods on BEV object detection (AP, %) on KITTI test set.

Method	Car			Pedestrian			Cyclist			Input Data
	Easy	Mod	Hard	Easy	Mod	Hard	Easy	Mod	Hard	
VoxelNet [29]	89.35	79.26	77.39	46.13	40.74	38.11	66.70	54.76	50.55	LIDAR
PointRCNN [22]	84.32	75.42	67.86	85.94	75.76	68.32	89.47	85.68	79.10	LIDAR
SECOND [31]	83.13	73.66	66.20	51.07	42.56	37.29	70.51	53.85	46.90	LIDAR
MV3D [7]	86.02	76.90	68.49	N/A	N/A	N/A	N/A	N/A	N/A	LIDAR + Image
AVOD [3]	86.80	85.44	77.73	42.51	35.24	33.97	63.66	47.74	46.55	LIDAR + Image
AVOD-FPN [3]	88.53	83.79	77.90	50.66	44.75	40.83	62.39	52.02	47.87	LIDAR + Image
F-PointNet [35]	88.70	84.00	75.33	58.09	50.22	47.20	75.38	61.96	54.68	LIDAR + Image
MMF [10]	93.67	88.21	81.99	N/A	N/A	N/A	N/A	N/A	N/A	LIDAR + Image
CLOCs_SecCas [8]	91.16	88.23	82.63	N/A	N/A	N/A	N/A	N/A	N/A	LIDAR + Image
Our	94.10	88.62	82.33	60.92	52.58	49.14	77.65	63.26	56.42	LIDAR + Image
